# P-191. Detection of Hearing Loss by Formal Audiological Screening Test in Pediatric and Adult Acute Meningitis: A Global Systematic Review and Meta-analysis

**DOI:** 10.1093/ofid/ofaf695.414

**Published:** 2026-01-11

**Authors:** Luisa F Alviz, Carla Y Kim, Ana C Benevides-Tadinac, Jackson A Roberts, Lauren E Monette, Caroline E Harrer, Francisco J Varela, Soonmyung A Hwang, Blen M Gebresilassie, Pilar Balcarce, Manya Prasad, John Usseglio, Kiran T Thakur

**Affiliations:** Columbia University, Bogotá, Distrito Capital de Bogota, Colombia; Columbia university, New York, New York; University of South Carolina, West Columbia, South Carolina; Columbia University, Bogotá, Distrito Capital de Bogota, Colombia; Columbia University, Bogotá, Distrito Capital de Bogota, Colombia; The Rockefeller University, New York, New York; Fleni institute, Buenos Aires, Buenos Aires, Argentina; Icahn School of Medicine at Mount Sinai, New York, New York; Columbia University, Bogotá, Distrito Capital de Bogota, Colombia; Fleni institute, Buenos Aires, Buenos Aires, Argentina; All India Institute of Medical Sciences, New Delhi, Delhi, India; Columbia University, Bogotá, Distrito Capital de Bogota, Colombia; Columbia University Irving Medical Center, Orangeburg, NY

## Abstract

**Background:**

Acute community-acquired bacterial meningitis remains a significant global health concern with significant mortality and morbidity, including neurological sequelae such as sensorineural hearing loss (SNHL). Early detection of meningitis-associated SNHL mitigates permanent deafness and poor outcomes. This systematic review evaluates the optimal timing of formal audiological testing in relation to the diagnosis of acute meningitis in adults and children.

**Methods:**

A literature search was conducted across Ovid Medline, Elsevier Embase, and Cochrane databases. Studies reporting the time frames for hearing loss (HL) detection secondary to acute meningitis using formal audiological tests were included. Data were analyzed descriptively for continuous and categorical variables. A meta-analysis calculated the pooled prevalence of outcomes, with subgroup analyses stratified by the time frame of audiological screening.

**Results:**

A total of 41 studies were included, with 8,105 meningitis patients comprising 1,397 (17.2%) adults and 6,708 (82.8%) children. In adults, most audiological screenings occurred post-discharge, yet the proportion of hearing loss diagnoses was higher before discharge than after (45.5% vs. 42.5%). Similarly, in children, more screenings were administered post-discharge compared to before (3,340 vs. 1,975), but HL diagnoses were more frequent before discharge. The pooled prevalence of HL diagnoses during hospitalization or at discharge was 30.4% (95% CI 22.9-38%), compared to 22.9% (95% CI 12.6-33.1%) within one-month post-discharge, 20.3% (95% CI 8.8-31.9%) between 30 to 60 days post discharge, 22.7% (95% CI 12.1-33.4%) between 60-180 days post-discharge, and 10.8% (95% CI 10.8-15.7%) after 180 days of discharge.

**Conclusion:**

The considerable variability in the time frame of audiological test administrations following an acute meningitis episode highlights the need for standardized auditory evaluations after meningitis diagnosis. Our findings suggest that early implementation of SNHL screening, particularly before discharge, should be prioritized alongside proper follow-up screening in children and adults.

**Disclosures:**

Kiran T. Thakur, MD FAAN, Center for Disease Control: Grant/Research Support|Delve Bio: Advisor/Consultant|World Health Organization: Advisor/Consultant

